# Identification of the Cellular Tipping Point in the Inflammation Model of LPS-Induced RAW264.7 Macrophages Through Raman Spectroscopy and the Dynamical Network Biomarker Theory

**DOI:** 10.3390/molecules30040920

**Published:** 2025-02-17

**Authors:** Akinori Taketani, Shota Koshiyama, Takayuki Haruki, Shota Yonezawa, Jun Tahara, Moe Yamazaki, Yusuke Oshima, Akinori Wada, Tsutomu Sato, Keiichi Koizumi, Isao Kitajima, Shigeru Saito

**Affiliations:** 1 Research Center for Pre-Disease Science, University of Toyama, Toyama 930-8555, Japanyonezawa@cts.u-toyama.ac.jp (S.Y.); s30saito@med.u-toyama.ac.jp (S.S.); 2 Division of Presymptomatic Disease, Institute of Natural Medicine, University of Toyama, Toyama 930-0194, Japan; 3 Faculty of Sustainable Design, University of Toyama, Toyama 930-8555, Japan; 4 Graduate School of Science and Engineering, University of Toyama, Toyama 930-8555, Japan; 5 Faculty of Engineering, University of Toyama, Toyama 930-8555, Japan; 6 Department of Hematology, Faculty of Medicine, Academic Assembly, University of Toyama, Toyama 930-8555, Japantsutomus@med.u-toyama.ac.jp (T.S.)

**Keywords:** DNB theory, inflammation model, Raman spectroscopy, tipping point, tryptophan

## Abstract

Raman spectroscopy is a non-destructive spectroscopic technique that provides complex molecular information. It is used to examine the physiological and pathological responses of living cells, such as differentiation, malignancy, and inflammation. The responses of two cellular states, initial and full-blown inflammation, have mainly been investigated using a comparative analysis with Raman spectra. However, the tipping point of the inflammatory state transition remains unclear. Therefore, the present study attempted to identify the tipping point of inflammation using a cell model. We stimulated RAW264.7 mouse macrophages with lipopolysaccharide (LPS) and continuously collected Raman spectra every 2 h for 24 h from the initial and full-blown inflammation states. A Partial Least Squares analysis and Principal Component Analysis—Linear Discriminant Analysis predicted the tipping point as 14 h after the LPS stimulation. In addition, a Dynamical Network Biomarker (DNB) analysis, identifying the tipping point of a state transition in various phenomena, indicated that the tipping point was 14 h and identified tryptophan as a biomarker. The results of a multivariate analysis and DNB analysis show the cellular tipping point.

## 1. Introduction

Raman spectroscopy is a vibrational spectroscopic technique that collects information on molecular structures and chemical binding from samples in a non-destructive and label-free manner [1,2,3]. Due to its minimal invasiveness, the application of Raman spectroscopy to medical on-site diagnostics is expected. Many optical tools have been developed and Raman microscopy approaches are used to examine living cells [4,5]. Analyses of complex Raman spectra have been established using a combination of multivariate analytical methods, such as a Principal Component Analysis (PCA). Raman spectroscopy is a powerful tool for identifying cell differentiation and malignant cells [6,7]. Regarding cells involved in inflammation, previous studies used surface-enhanced Raman spectroscopy to demonstrate the effects of lipopolysaccharide (LPS) on macrophages [8], while others employed Raman spectroscopy to identify M1 and M2 polarizations in macrophages [9]. Inflammation is a crucial response that removes external invaders, such as bacteria. While this response is necessary for maintaining balance, chronic inflammation is a significant health concern [10,11]. However, the tipping point of a state transition in the inflammatory response remains unclear.

A state transition from stable state A to B occurs through an unstable state, which is referred to as the tipping point. Fluctuations in measured variables increase during state transitions in biological phenomena, as shown in the bottom of the energy potential in a dynamic system [12,13]. Chen and Aihara et al. constructed the Dynamical Network Biomarker (DNB) theory by introducing the bifurcation theory for the detection of a critical transition state before the onset of a disease state [12,14]. The DNB theory detects the early warning signals of state transitions, specifically unstable states, in complex networks of biological systems. This theory has been applied to the gene expression profiles of diseases, revealing tipping points for various pre-disease states [12,14,15,16,17,18,19,20,21,22]. The DNB theory allowed for the prediction and detection of the tipping point at which hepatocellular carcinoma metastasizes to the lungs, demonstrating the utility of this theory for the prevention of disease progression [16]. The application of the DNB theory to Raman spectroscopy has started in various fields. In the non-clinical research, Haruki et al. applied the DNB theory to the Raman spectra of the mouse T-cell activation process, in which naïve cells become fully activated [23]. In the clinical research, Yonezawa et al. applied the DNB theory and an energy landscape analysis to the Raman spectra of multiple myeloma and monoclonal gammopathy of undetermined significance [24]. However, in the case of human clinical data, difficulties have been associated with the preparation of control groups, and the number of points was also limited. The use of the DNB theory in Raman spectroscopy requires a suitable model.

In the present study, we aimed to identify the tipping point of the inflammatory response using an inflammatory cell model and the application of Raman spectroscopy and the DNB theory. We used a cellular inflammation model by adding LPS to RAW264.7 cells. Traditionally, RAW264.7 cells stimulated with LPS have been analyzed for their expression of pro-inflammatory cytokines, including TNF-α, IL-6, and NO, using methods such as RT-qPCR, Western blotting, and ELISA [25,26]. Raman spectroscopy provides a unique benefit by allowing real-time observations of direct molecular changes in cells. The present results demonstrate our understanding of inflammatory cells and show that the combined Raman spectroscopy and DNB theory analysis accurately captures the tipping point. Further studies are needed to establish whether interventions at tipping points are capable of suppressing the progression of inflammation.

## 2. Results

**Figure 1:**
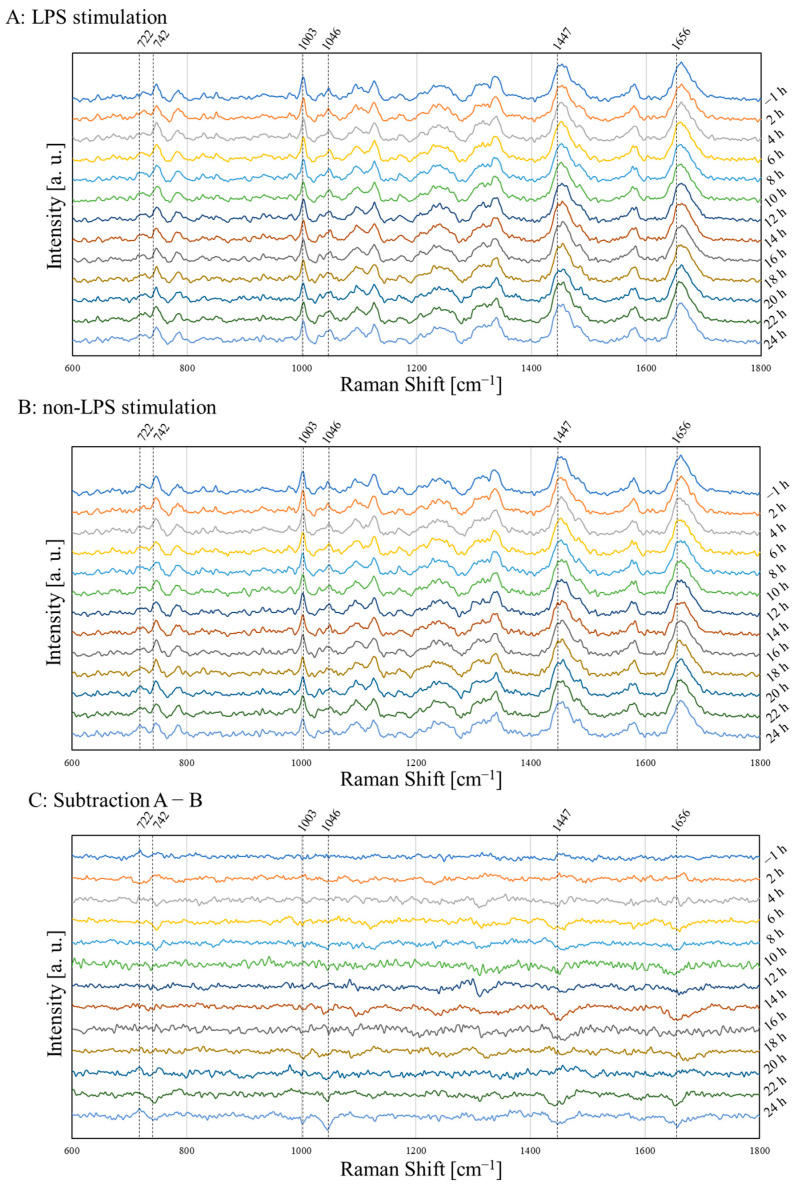
Raman spectra of LPS stimulation and non-stimulation groups: (**A**) the LPS stimulation group; (**B**) the non-stimulation group; (**C**) the subtracted Raman spectra of (**A**,**B**).

Figure 1 shows the Raman spectra of the LPS stimulation and non-stimulation groups from −1 h to 24 h. Figure 1A represents the stimulated group, Figure 1B the non-stimulation group, and Figure 1C the subtracted data of Figure 1A,B. The mean value is the average of 30 spectra obtained from 30 cells at each time in three similar experiments. The observed differences between the stimulated and non-stimulation groups demonstrate that this Raman device possesses sufficient sensitivity for precise measurements. Table 1 shows characteristic Raman bands changed by the stimulation in Figure 1C [27,28,29,30,31]. Inflammatory cells changed at 722, 742, 1003, 1046, 1447, and 1656 cm^−1^ Raman shifts to induce the LPS stimulation. DNA bands changed up and down after 20 h. Lipid and protein bands decreased at 14, 22, and 24 h. A reduction in the proline band was observed from 20 to 24 h.

**Figure 2:**
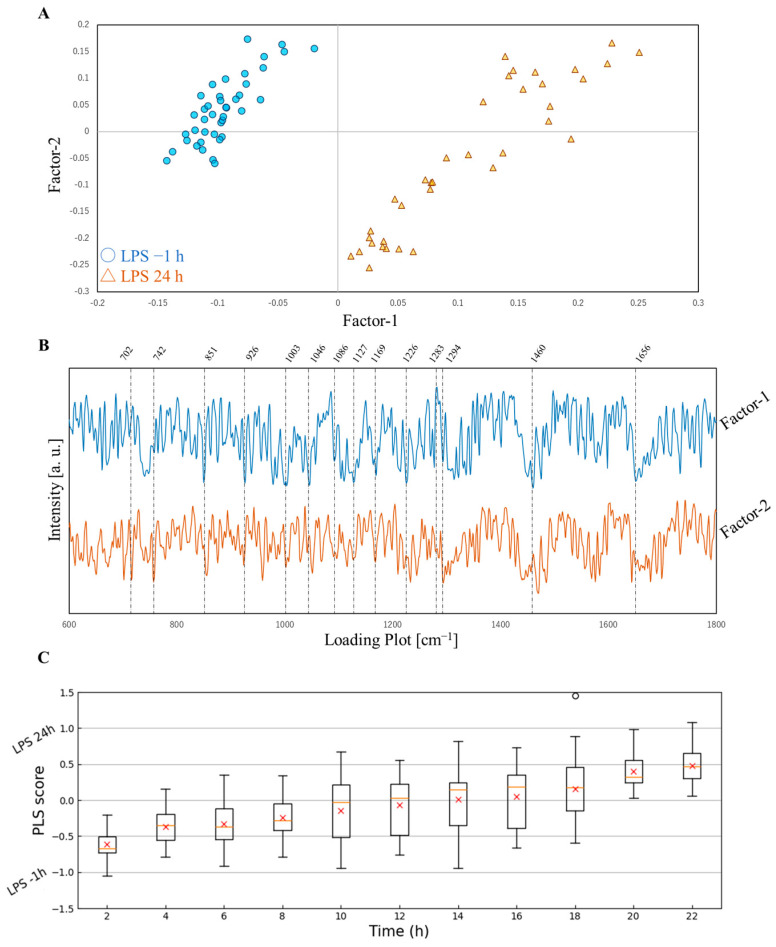
PLS analysis of Raman spectra −1 h and 24 h after the LPS stimulation. (**A**): PLS score plot of Factor-1 and Factor-2; (**B**): loading plot of Factor-1 and Factor-2; (**C**): box plots of 2 to 22 h predictions between −1 h and 24 h using the PLS model. Boxes represent the interquartile range (IQR). Whiskers extended 1.5× the IQR. Circle plots indicate outliers, while red cross plots represent averages. Orange horizontal lines within boxes show the median.

A PLS analysis of the acquired Raman spectra is shown in Figure 2. In Figure 2A, the score plots of Factor-1 and Factor-2 indicate a clear division into two categories for the time points of −1 h and 24 h after the LPS stimulation, as confirmed by Factor-1. The contributions of Factor-1 and Factor-2 were approximately 80 and 20%, respectively. Factor-1 played a dominant role in the classification of the −1 h and 24 h groups. Figure 2B shows a loading plot of Factor-1 and Factor-2, with several characteristic bands being identified. Using Factor-1 and Factor-2 obtained from the PLS analysis, we developed a model to classify −1 h and 24 h after the LPS stimulation. The results obtained are shown in Figure 2C, illustrating sample forecasts for classifying the remaining 2 to 22 h. The model classified a PLS score near to −1 as −1 h and a score near to 1 as 24 h. The corresponding bands are shown in Table 2 [27,29,30,31,32,33,34,35]. In Figure 2C, 742, 1003, 1046, and 1656 cm^−1^ were the same band in the subtracted spectra. The analysis indicated that a structural change in inflammatory cells from −1 h to 24 h was captured by PLS analysis Factors-1 and -2. The classification changed from −1 h to 24 h as each hour passed. Data variance increased from 10 h, peaked at 14 h, and then gradually converged toward 24 h as the time progressed.

**Figure 3:**
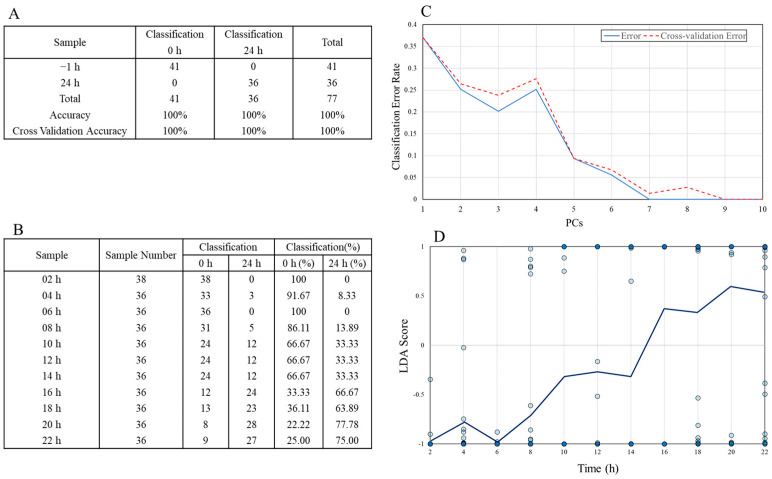
PCA-LDA analysis of discriminant results from 2 to 22 h after the stimulation using −1 h and 24 h models. (**A**): Results of model creation for −1 and 24 h; (**B**): results of adaptation of the discriminant model from 2 to 22 h; (**C**): number of PCs used and model error rates; (**D**): score plots for model application results for each hour.

The results of the PCA-LDA of discriminant results from 2 to 22 h after stimulation using −1 h and 24 h models are shown in Figure 3. The models were created using PCA data from LPS −1 at 24 h, and a discriminant analysis of 2–22 h data was performed. The accuracy results of the discriminant model (A) show 100% discrimination accuracy for both −1 and 24 h. Discriminant results confirm that this group was divided into −1 h and 24 h after 14 h. The number of PCs used versus the model error was plotted to select how many PCs to use when creating the discriminant model (C). Eight PCs were used in the analysis, up to PC10, excluding PC4 and PC8, which reduced the discriminant accuracy. These results confirm that more decisions were made 16 h after the LPS stimulation toward the 24 h side.

**Figure 4:**
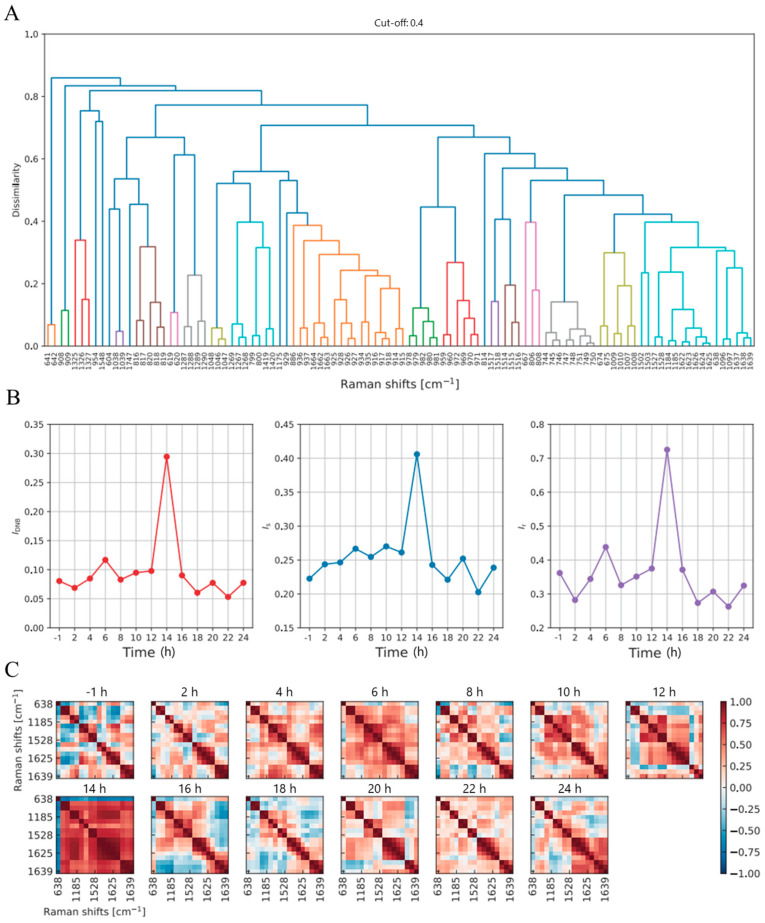
Results of the DNB analysis of the LPS stimulation: (**A**): a dendrogram of highly fluctuating Raman shifts; (**B**): (**left**) DNB scores, (**center**) the average standard deviation, and (**right**) the average correlation strength; (**C**): a heat map of correlations among the DNB group (cyan-colored part).

**Figure 5:**
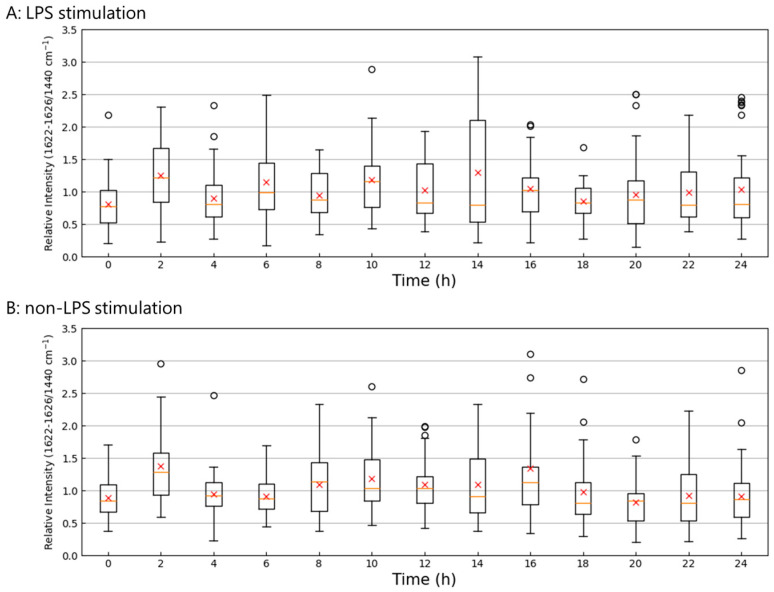
Time series of the relative intensity of the tryptophan band. (**A**): LPS-stimulation groups; (**B**): non-LPS stimulation groups. Boxes represent the IQR. Whiskers extended 1.5× the IQR. Circle plots indicate outliers, while red cross plots represent averages. Orange horizontal lines within the boxes show the median.

The results of the DNB analysis of the LPS stimulation are shown in Figure 4. Highly fluctuating Raman shifts through the F-test between the control and experimental groups were arranged along the horizontal axis in Figure 4A. The two large clusters with a size of 17 Raman shifts were extracted as the DNB candidate group shown in the orange- and cyan-colored parts. The cyan-colored DNB candidate group only showed a peak in the time development of DNB scores in Figure 4B (left). The average standard deviation and correlation strength also showed a peak at 14 h (see Figure 5B (center) and (right)). In contrast, the orange-colored DNB candidate did not show a peak. Therefore, the tipping point of inflammation was identified as 14 h and the shifts at 638 (methionine), 1096–1099 (PO^2−^ in DNA and C–C lipids), 1184–1185 (DNA), 1502–1503 (no specific assignments), 1527–1528 (carotenoid), 1622–1626 (tryptophan), and 1638–1639 (Amide I) cm^−1^ were identified as DNB Raman shifts [31,34,36,37,38]. The corresponding bands are summarized in Table 3. Figure 4C shows a heatmap of the correlation among DNB groups at each time point. The correlation between DNB Raman shifts became strong at 14 h, the tipping point of inflammation.

Based on the results shown in Figure 4, the presence of tryptophan is confirmed. Figure 5 shows temporal changes in the tryptophan levels. To analyze the data, the intensity of the tryptophan band at a range of 1622–1626 cm^−1^ was normalized against that at 1440 cm^−1^. The results obtained indicate that, while the amount of tryptophan fluctuates over time, its values remain within a consistent range, exhibiting no significant deviations as time progresses. In the group stimulated with LPS, a notable variation in the tryptophan band was observed 14 h after the stimulation.

## 3. Discussion

Marked differences were observed in the subtracted spectra between inflammation (LPS) and non-inflammation (non-LPS) (Figure 1C). The changes observed in proteins, DNA, and lipids in the Raman spectra may reflect inflammation-induced changes in cell morphology. The decrease observed in proline is important to note. Changes in proline were also confirmed in PLS analysis factor loadings (Figure 2B). Cells polarized into M2 macrophages convert arginine to proline in the process of wound healing; therefore, proline is regarded as an M2 marker [39,40]. In the present study, LPS-stimulated M1 polarization was ongoing, whereas M2 polarization was not. Therefore, the decrease in proline, an M2 marker, suggests that the inflammatory response of the full-blown inflammation state, M1 polarization, accelerated 20 h after the LPS stimulation.

The present results also demonstrate that the PLS prediction model effectively classifies the initial and full-blown inflammation states. According to the non-crossing of the zero axis of box plot distributions, 2 h may be classified as LPS −1 h, and 20 and 22 h as LPS −24 h (Figure 2C). Variance increased at 10 to 18 h and converged in the prediction model. With large fluctuations, maximum variance was observed at 14 h, resulting in the tipping point at 14 h. The results of the PLS analysis indicate that RAW264.7 cells fluctuate during the transition from the initial and full-blown inflammation states.

Furthermore, we introduced PCA-LDA methods to confirm the results of the PLS analysis. The results of PCA-LDA in Figure 3 indicate a clear shift from the initial to full-blown inflammation states between 14 and 16 h. This result agrees with the tipping point at 14 h identified by the PLS analysis. Therefore, PCA-LDA supported the results of the PLS analysis. However, it was difficult to identify biomarkers indicating the tipping point of inflammation. Therefore, we investigated biomarkers that indicate the tipping point of inflammation using the DNB analysis. A peak in the DNB score corresponded to the tipping points observed in the PLS analysis and PCA-LDA.

The Raman shifts associated with tryptophan generally show a positive correlation in Figure 4C. In contrast, the Raman shift associated with methionine uniquely displayed a negative correlation with other Raman shifts. Methionine plays a crucial role in immune function [41]. However, its Raman shift was only observed at 638 cm^−1^. Carotenoids, assigned in 1527–1528 cm^−1^, interact with the NF-κB pathway and, thus, inhibit the downstream production of inflammatory cytokines [42]. In contrast, Raman bands attributed to tryptophan were identified at multiple wavelengths, specifically 1622–1626 cm^−1^. The Raman bands of tryptophan were absent in the subtracted spectra and PLS analysis; however, tryptophan was identified as a biomarker of inflammation at the tipping point through the DNB analysis. These results suggest that tryptophan plays a significant role during the critical tipping point.

Tryptophan is metabolized during inflammation to produce various metabolites, such as kynurenine and picolinic acid. Kynurenine activates aryl hydrocarbon receptors and contributes to the anti-inflammatory effects of macrophages [43]. Picolinic acid has been shown to promote inflammatory responses by inducing the expression of specific chemokines, such as macrophage inflammatory protein (MIP)-1α and MIP-β in macrophages [44]. 3-Hydroxyanthranilic acid (3-HAA), an intermediate metabolite in the tryptophan metabolic pathway, is known for its antioxidant and immunomodulatory properties. Previous studies reported that supplementation with 3-HAA in Caenorhabditis elegans and mice suppressed oxidative stress and the production of pro-inflammatory cytokines, leading to lifespan extension [45]. The metabolism of tryptophan significantly affects the polarization of M1 macrophages [46]. As shown in Figure 5, no decrease was detected in tryptophan levels, which may have been due to its ample availability in the culture medium. Fluctuations in tryptophan levels suggest that significant changes in its metabolism may have occurred at transitional points. The degradation of tryptophan plays a critical role in inflammation and is reportedly involved in 13 types of chronic inflammatory diseases [47]. Therefore, targeting tryptophan dynamics during transitional states may provide a promising approach for treating inflammatory disorders.

The fluctuations observed in tryptophan at the tipping point indicate that the modulation of tryptophan metabolism affected these fluctuations in inflammation. Future studies that focus on removing tryptophan from the culture medium and examining whether this abolishes the inflammatory transition point in macrophages are warranted. The administration of tryptophan metabolism inhibitors, specifically indoleamine 2,3-dioxygenase and tryptophan 2,3-dioxygenase inhibitors, has potential as a therapeutic intervention at the tipping point of inflammation [48,49]. The administration of these tryptophan metabolism inhibitors before the tipping point is crucial for stabilizing fluctuations in the inflammatory response. This approach may pave the way for early therapeutic interventions during the inflammatory response.

Raman spectroscopy is a rapid and minimally invasive technique that captures complex molecular information from samples. However, due to inherently weak signals, it is necessary to validate the accuracy of the Raman spectra obtained in the present study. Other spectroscopic techniques, such as nuclear magnetic resonance, face challenges in measuring living cells, and the introduction of fluorescent probes raises concerns regarding potential impacts on cellular expression systems [50,51]. In consideration of these limitations and the time constraints of the experiment, we opted to use Raman spectroscopy exclusively in the present study.

Regarding the reliability of Raman spectra, the PLS analysis revealed one outlier. Nevertheless, the data points at 2 h and 22 h were clearly distributed before and after the onset of inflammation, respectively, indicating that data variation is acceptable. We intend to confirm the transition points identified in the present study through biological approaches, such as a gene expression analysis, in future research.

## 4. Materials and Methods

### 4.1. Raman Microscope Devices

Cells were observed under a 60× oil immersion objective (Apo TIRF: Nikon, Tokyo, Japan) on a microscope (ECLIPSE Ti: Nikon, Tokyo, Japan) equipped with a motorized stage. A diode-pumped solid-state laser (Cobolt Samba 150, 532 nm, Stockholm, Sweden) was introduced into the microscope as an excitation source, and the intensity of the laser was adjusted between 0.2 and 20 mW at the sample point. The spectroscopic detector was a cooled CCD camera (BI-DD Cooled CCD, Andor, Belfast, Northern Ireland). The system was invented to measure living cells seeded in dishes with quartz bottoms. In one trial, Raman spectra were collected over 30 s with a 2 s integration time from 10 random cells. We confirmed that the Raman spectra obtained under these measurement conditions were sufficient to observe M1 polarization induced by inflammation [52].

### 4.2. Cell Treatment

We used an inflammation cell model in which LPS was added to mouse macrophages (RAW264.7 cells). RAW264.7 (TIB-71, ATCC) cells were cultured in Dulbecco’s Modified Eagle Medium (D-MEM, WAKO) supplemented with 10% (*v*/*v*) heat-inactivated fetal bovine serum (ATCC), 100 U/mL penicillin (Meiji, Tokyo, Japan), and 100 μg/mL streptomycin (Meiji, Japan).

**Figure 6:**
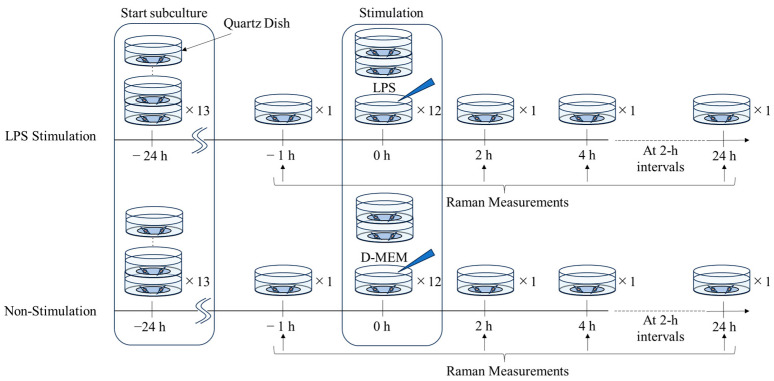
The Raw264.7 cell culture and Raman measurement timeline. Thirteen samples each from the LPS-stimulated and non-stimulated groups were prepared and passaged 24 h prior to the start of the stimulation. Raman measurements were taken 1 h before the LPS stimulation and then at 2-h intervals for 24 h after the stimulation, for a total of 13 measurements.

Raman measurements were performed to follow changes in cells. RAW264.7 cells were seeded at a density of 1 × 10^5^ cells on a quartz dish (SF-S-D12, Fine Plus International Ltd., Kyoto, Japan). Twenty-four hours after feeding, LPS (SIGMA-ALDRICH, Saint Louis, MO, USA) at 100 ng/mL dissolved in Dulbecco’s phosphate-buffered saline (−) (Nissui, Tokyo, Japan) was added to RAW264.7 cells. We initially considered using an LPS concentration of 10 ng/mL. However, a previous study reported that the inflammatory response may vary at an LPS concentration of 10 ng/mL [53]. To ensure a consistent inflammatory response in our experiment, we increased the concentration of LPS to 100 ng/mL, as supported by previous findings [54,55]. We established non-stimulation control groups using D-MEM instead of LPS. We initially intended to use a single dish for Raman spectroscopy measurements. However, due to the considerable impact of measurements on cells, we used 13 individual dishes. We conducted cell passages 24 h before measurements. Raman measurements began 1 h before the stimulation (−1 h) and were taken at 2 h intervals for 24 h after the stimulation because of the high time resolution and sampling limitation. The excitation laser was focused on nuclei in cells. We collected a single Raman spectrum per cell each time. This experiment was repeated three times, and the Raman data from all three measurements were combined for analyses (Figure 6).

### 4.3. Raman Data Preprocessing

Since Raman spectra contain many noise components and require correct pre-processing, we used Python for spectral pre-processing, which subtracted culture media and quartz spectra from the Raman spectra. The Python module skimage restoration rolling ball was used to calculate rolling ball baseline corrections with a radius of 50 in the wavenumber range of 1850–550 cm^−1^. Rama spectra were then smoothed using a Savitzky–Golay filter with a window length (number of data used for approximation) of 7 and polynomial degree of 3 using the Python module scipy signal savgol filter. Intensity corrections were performed by dividing intensity by the average intensity of the entire spectrum.

### 4.4. Partial Least Squares (PLS) Analysis

A calibration curve model was created for a PLS analysis. The dependent variable defined −1 h as −1 and 24 h as 1 after the stimulation. The remaining Raman data obtained from 2 to 22 h were predicted using the calibration curve to establish whether they corresponded to −1 h or 24 h. Cross-validation was used to build the model, and the optimal number of factors was selected. The number of factors in the PLS analysis was also set by cross-validation. The PLS analysis was used from Factors 1 to 2.

### 4.5. PCA-Linear Discriminant Analysis (LDA)

LDA is a method for distinguishing between different data groups by creating a linear model that separates them in advance. In this case, PCA was initially performed on all Raman data to extract the components needed for discrimination. LDA was then conducted based on the PC scores obtained. Models were initially created using the PC 1–10 score data from the PCA of LPS −1 at 24 h Raman spectra. The number of principal components used in the LDA model was selected based on the error score of cross-validation. The discriminant analysis model was used and the data were obtained in the range of 2–22 h after the stimulation.

### 4.6. DNB Analysis

A DNB analysis detects an early warning signal at the tipping point of a state transition. The basic procedure for the DNB analysis is listed below.

1. Preprocessing;
2. The F-test to evaluate fluctuations between the experimental and control groups;
3. Correlations to assess the relationships between fluctuating variables;
4. Clustering to define DNB candidates;
5. DNB scores to identify the transition state and DNB elements.

We defined the data for each time point as the matrix *X* = $x_{ik}$ (*i* = 1, …, N, *k* = 1, …, K), where *i* and *k* are the indices of the Raman shifts (variables) and cells (samples), respectively. N is the total number of variables, 1201 Raman shifts, and K is the number of samples, 30 cells, at that time point. The mean and standard deviation of data are defined as $m_{i}\left(X\right)$ and $s_{i}\left(X\right)$, respectively.

We calculated the variance in each Raman shift between the experimental and control groups at each time point. A one-tailed F-test outputted highly fluctuating Raman shifts, and the Benjamini–Hochberg method was used to suppress the false discovery rate. The significance level, a modified *p*-value, was set at 0.05 for the hypothesis test. We also calculated the correlation $r_{ij}$ between i- and j-th highly fluctuating Raman shifts using the quantities of $m_{i}\left(X\right),m_{j}\left(X\right),s_{i}\left(X\right),$ and $s_{j}\left(X\right)$ and their dissimilarity, defined as $d=1-\left|r_{ij}\right|\;(0\leq d\leq 1)$. Hierarchical clustering based on this dissimilarity produced several clusters using a specific threshold, the cut-off value *d* = 0.4. The largest cluster and larger clusters became DNB candidate groups. In addition, we calculated the average standard deviation and correlation strength at each time point among DNB candidates. The DNB score is defined as the product of its average standard deviation ($I_{s}$) and correlation strength ($I_{r}$), $I_{DNB}=I_{s}\cdot \;I_{r}$. When the DNB score shows a peak in time, the peak time is identified as the tipping point, and their Raman shifts are selected as DNB Raman shifts. In contrast to previous studies, a control group without LPS was also prepared for an analysis over time and compared simultaneously from the stimulation.

## 5. Conclusions

In the inflammatory cell model with LPS added to RAW264.7 cells, the inflammatory tipping point was successfully detected using the DNB analysis and corresponded to that in a PLS analysis of Raman spectra. PCA-LDA also showed convergence to inflammation from the 14 h time point onward, indicating that the combined Raman spectroscopy and DNB analysis correctly captured the tipping point detected. The DNB analysis identified tryptophan as an inflammation biomarker at the tipping point. The ability to detect early warning signals at tipping points in inflammatory responses will provide novel insights for future studies on inflammatory reactions. Furthermore, tipping points identified by Raman spectroscopy and the DNB theory may serve as biomarkers and contribute to the suitable timing of therapeutic interventions against inflammation.

## Figures and Tables

**Table 1:** Assignment of subtracted Raman shifts in Figure 1.

Raman Shift [cm^−1^]	Assignment
722	DNA [27]
742	DNA [27]
1003	Phenylalanine [27]
1046	Proline [30]
1447	CH_2_ Lipid and Protein [29]
1656	C=C Fatty Acids and Amide I [28]

**Table 2:** Assignment of Raman shifts in the PLS loading plot in Figure 2B.

Raman Shift [cm^−1^]	Assignment	Factor-1	Factor-2
702	DNA [35]		-
742	DNA [27]	-	
851	Proline [34]	-	-
926	Proline [34]	-	-
1003	Phenylalanine [27]	-	-
1046	Proline [30]	-	-
1086	Lipid [34]	+	
1127	Protein [33]	-	
1169	Tyrosine [34]	-	-
1226	Amide III [32]	-	
1283	Amide III [32]	+	
1294	CH_2_ Deformation [29]		-
1460	CH_2_/CH_3_ Lipids and Collagen [34]	-	-
1656	C=C Fatty Acids and Amide I [28]	-	-

**Table 3:** Assignment of DNB Raman shifts in Figure 4A.

Raman Shift [cm^−1^]	Assignment
638	Methionine [36]
1096–1099	PO^2-^ in DNA and C-C Lipids [37]
1184–1185	DNA [38]
1502–1503	No Specific Assignments
1527–1528	Carotenoid [38]
1622–1626	Tryptophan [34]
1638–1639	Amide I [38]

## Data Availability

The data presented in this study are available on request from the corresponding authors.
